# Dermatological Presentations in the Adult Emergency Department: A Retrospective Audit of Frequency, Management, and Outcomes in a UK Tertiary Centre

**DOI:** 10.7759/cureus.92548

**Published:** 2025-09-17

**Authors:** Naheed Habibullah, Ria Gupta, Uday Mahajan, Huma Rahman, Vibhore Gupta

**Affiliations:** 1 Emergency Medicine, Queen Elizabeth Hospital, Birmingham, GBR; 2 Emergency Medicine, University of Birmingham, Birmingham, GBR; 3 Trauma and Orthopaedics, Queen Elizabeth Hospital, Birmingham, GBR; 4 Emergency Department, Queen Elizabeth Hospital, Birmingham, GBR

**Keywords:** british association of dermatologists, dermatology referral, emergency department attendances, nice guidelines, skin conditions

## Abstract

Background

Dermatological conditions frequently present to emergency departments (EDs), ranging from benign rashes to severe immunological emergencies. However, most ED clinicians receive little or no formal dermatology training, leading to variation in management and incomplete documentation, particularly of drug history and follow-up planning. In the absence of standardised referral pathways or local ED dermatology protocols, the quality of care is inconsistent and may not align with national standards.

Objective

To evaluate the frequency, diagnostic categories, management, and clinical outcomes of dermatology-related presentations to the adult ED. The audit aimed to assess compliance with national guidelines and determine whether the observed gaps highlight the need for a structured local dermatology protocol and standardised referral pathway.

Methods

A retrospective audit was conducted at a tertiary ED over a three-month period (March-June 2025). Data for 80 patients were extracted from electronic records. Variables included demographics, clinical diagnoses, treatments administered, investigations performed, comorbidities, and disposition outcomes. The audit was benchmarked against National Institute for Health and Care Excellence (NICE) and British Association of Dermatologists (BAD) guidelines.

Results

Of 80 patients, 60% were female, and the mean age was 42 years (range 8-95). Allergic reactions (23.8%) and cellulitis/abscess (20%) were the most common diagnoses. Blood tests were performed in 61.3% of patients; 60% received treatment. Antibiotics (20%) and antihistamines (12.5%) were commonly used. Dermatology follow-up occurred in only 6.3% of cases. A total of 21 patients (26.3%) were admitted, while 37.5% had no documented follow-up. Documentation of drug history was inconsistent.

Conclusion

Dermatological cases are a frequent and varied component of ED workload, but are not consistently managed according to guidelines. The findings highlight the impact of limited dermatology training on documentation and management, and demonstrate the need for a local dermatology protocol, improved education for ED staff, and standardised follow-up pathways.

## Introduction

Dermatological conditions account for a substantial number of presentations to emergency departments (EDs) worldwide, ranging from minor complaints to potentially life-threatening emergencies [1]. These include a spectrum of infections, allergic reactions, inflammatory dermatoses, and immunologically mediated syndromes such as Stevens-Johnson Syndrome (SJS) and Drug Reaction with Eosinophilia and Systemic Symptoms (DRESS) [2]. While many skin conditions seen in the ED are self-limiting, others require urgent recognition, prompt treatment, or specialist referral. Despite their frequency, dermatological complaints are often under-prioritised, misdiagnosed, or inconsistently managed in the emergency setting [3].

Studies have shown that emergency clinicians frequently lack formal training in dermatology, which contributes to variation in clinical assessment, treatment decisions, and follow-up arrangements [4]. Moreover, dermatological presentations are rarely managed in direct collaboration with dermatology services, leading to potential gaps in care continuity [5]. In the UK, national guidelines, such as those from the National Institute for Health and Care Excellence (NICE) and the British Association of Dermatologists (BAD), provide clear benchmarks for the management of skin-related emergencies, including the timely administration of antibiotics in suspected cellulitis and appropriate documentation in allergic reactions; yet, local compliance with these standards remains uncertain [6,7]. This gap formed the rationale for conducting the present audit.

Accordingly, this retrospective audit was conducted to evaluate the frequency, diagnostic categories, investigation and treatment practices, and clinical outcomes of dermatology-related presentations to a tertiary adult ED. The findings were intended to assess compliance with national standards and to identify whether there is a need for local protocols, enhanced education, and standardised referral pathways.

## Materials and methods

A retrospective audit was conducted at a tertiary ED over a three-month period (March-June 2025). This timeframe was chosen pragmatically to align with the hospital’s standard audit cycle and to ensure the feasibility of manual data extraction, while still capturing a representative sample of dermatology-related presentations across the ED. All consecutive eligible patients during this period were included; therefore, no prior sample size calculation was undertaken.

Eligible cases were identified through the ED electronic patient record system using presenting complaint and diagnosis fields containing dermatology-related terms. International Classification of Diseases (ICD-10) codes were not used for case identification. Data were extracted manually into a standardised proforma by two investigators and cross-checked for accuracy. Variables collected included demographics, clinical diagnoses, treatments administered, investigations performed, comorbidities, and disposition outcomes. Missing data were coded as “not documented” and excluded from statistical analysis where appropriate.

The audit was benchmarked against relevant standards from NICE and BAD, including timely antibiotic administration in suspected cellulitis and appropriate documentation in allergic reactions.

Statistical analysis was performed using chi-square tests to assess associations between categorical variables (e.g., diagnosis and treatment, investigations and follow-up). This test was selected, as it is appropriate for assessing relationships between categorical data. Other tests were not applied, as the dataset did not contain continuous variables requiring parametric analysis. A significance level of p < 0.05 was used.

This project was registered and approved as a local clinical audit within the hospital’s governance framework and, therefore, did not require formal research ethics committee approval.

## Results

A total of 80 patients were included in the final analysis after excluding 59 who were referred directly to urgent care. The mean age was 41.9 years (SD ± 20.5), ranging from 8 to 95 years. The largest age group was 21-40 years with 36 patients (45%), followed by 41-60 years with 25 patients (31.3%). Females formed the majority with 48 patients (60%), reflecting a modest predominance of women among dermatology presentations.

**Figure 1:**
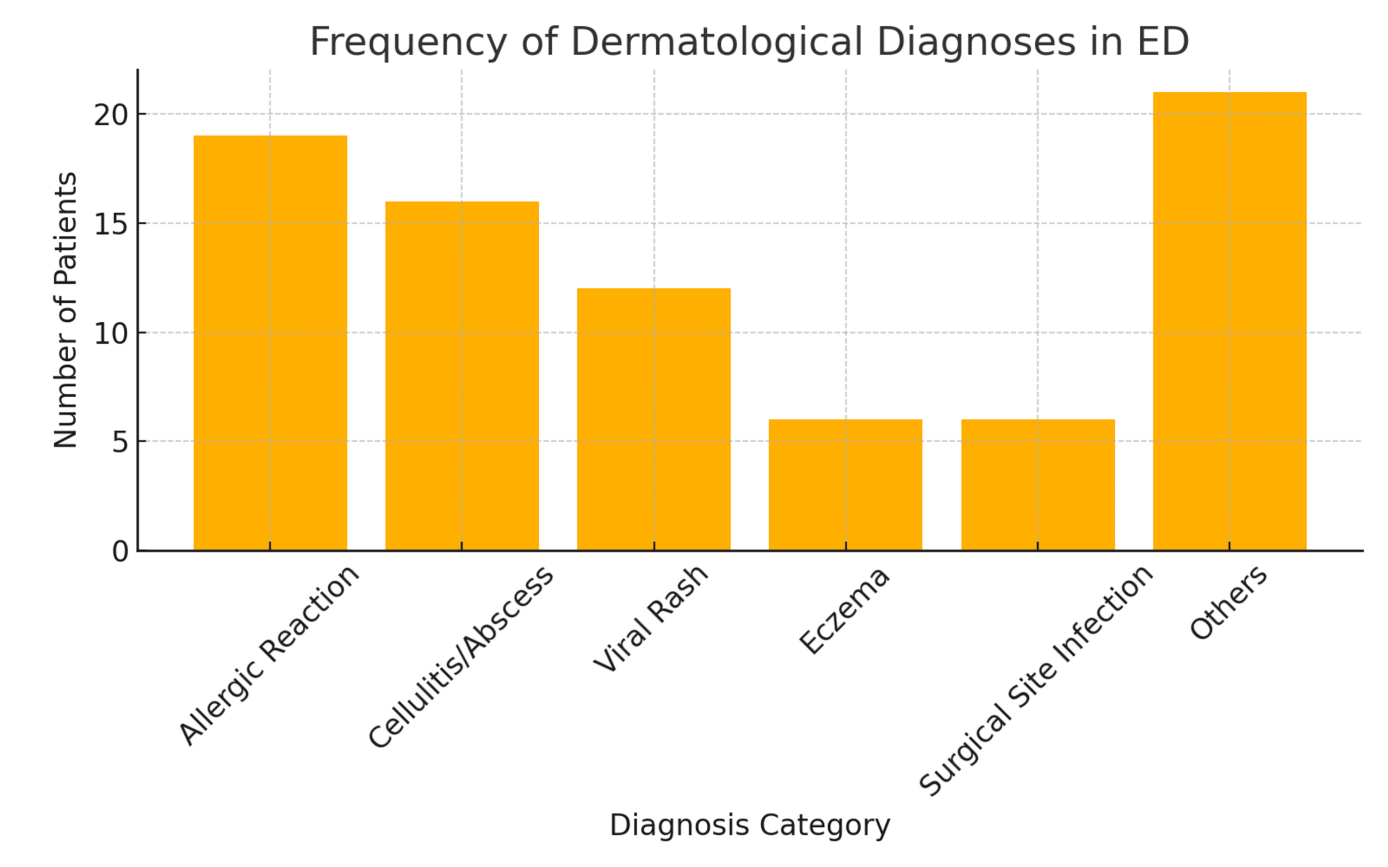
Distribution of the most frequent diagnoses in the cohort

Allergic reactions and cellulitis or abscesses were the most frequent diagnoses, together accounting for nearly half of all presentations. Viral rashes and eczema were also common, while a range of other conditions, such as drug eruptions, contact dermatitis, and fungal infections, made up the remainder. This distribution highlights the predominance of acute inflammatory and infectious dermatoses in the emergency setting (Figure 1).

Most patients underwent blood tests, and 60% received some form of treatment (Table 1). Antibiotics were the most commonly prescribed medication, followed by antihistamines and corticosteroids. However, a substantial proportion of patients (40%) received no documented therapy in the ED. Comorbidities were documented in 30 patients (37.5%), diabetes in 12 patients (15%), and asthma/chronic obstructive pulmonary disease (COPD) in 7 patients (8.8%). Eczema was recorded in 6 patients (7.5%) and immunosuppression in 4 patients (5%). The majority of patients, 50 (62.5%), had no recorded comorbidity.

**Table 1:** Investigations and management "Patients Treated" refers to those who received any therapeutic intervention in the ED.

Category	Details
Investigations Performed	Blood tests in 49 patients (61.3%)
Patients Treated	48 patients (60%)
Antibiotics Used	16 patients (20%)
Antihistamines Used	10 patients (12.5%)
Combination (Antihistamine + Prednisolone)	7 patients (8.8%)
Patients Referred to Dermatology	5 patients (6.3%)
No Documented Follow-Up	30 patients (37.5%)
Patients Admitted	21 patients (26.3%)

Clinical outcomes were coded as hospital admission, discharge with follow-up arranged (GP or dermatology), discharge without follow-up, referral to another speciality, or self-discharge. In our cohort, 21 patients (26.3%) required hospital admission, most often for cellulitis, abscesses, or systemic allergic reactions. A significant concern was the lack of documented follow-up in 30 patients (37.5%). When arranged, follow-up most frequently involved the general practitioner in 21 patients (26.3%), while only 5 patients (6.3%) were referred to dermatology. Two patients (2.5%) self-discharged, and 1 patient (1.3%) was referred to the maxillofacial team.

Chi-square testing revealed no significant associations between treatment and patient age (p = 0.615), gender (p = 0.402), diagnosis category (p = 0.327), or symptom duration (p = 0.256). Symptom duration was also not associated with admission (p = 0.466). However, there was a significant association between the performance of investigations and the likelihood of documented follow-up (χ² = 25.006, p < 0.001). This suggests that patients who underwent investigations were more likely to receive structured aftercare. Full results are presented in Table 2.

**Table 2:** Statistical analysis results The Pearson chi-square test was used. Statistical significance set at p < 0.05.

Variable Comparison	Test Used	χ² Value	p-value
Diagnosis vs Treatment	Chi-square	16.865	0.327
Age group vs Treatment	Chi-square	2.667	0.615
Gender vs Treatment	Chi-square	0.703	0.402
Symptom duration vs Treatment	Chi-square	4.049	0.256
Symptom duration vs Admission	Chi-square	2.551	0.466
Investigations vs Follow-up	Chi-square	25.006	<0.001

## Discussion

This audit highlights that dermatological conditions form a significant and diverse component of presentations to the adult emergency department (ED). Over the three-month audit period, a substantial number of patients presented with skin-related complaints, with allergic reactions and skin infections, such as cellulitis and abscesses, being the most frequently diagnosed conditions. These findings are consistent with previous studies, which have similarly identified infections, urticaria, and non-specific rashes as common reasons for ED dermatology consultations [1,8]. Despite the frequency of dermatological presentations, the audit reveals considerable variation in management and documentation practices. Notably, 40% of patients received no treatment, and in many cases, no investigations were performed. This may reflect a lack of structured guidance or limited dermatology training among ED clinicians. The underutilization of dermatology referrals, with only 6.3% of patients being formally referred, suggests that many cases are either under-recognised in terms of their complexity or are deemed self-limiting without specialist input. Previous literature has emphasised the benefit of dermatology liaison or teledermatology systems in improving triage accuracy and reducing unnecessary admissions--a model that could be considered in this setting [9-11].

A concerning observation is the high rate of incomplete follow-up. Over one-third of patients had no documented follow-up plan, which raises questions about continuity of care and the potential for missed diagnoses or treatment failures. The NICE and BAD guidelines emphasise timely treatment (e.g., antibiotics within one hour for cellulitis) and detailed documentation, particularly in suspected allergic reactions or drug-induced rashes [6,7]. This audit indicates that adherence to such standards is inconsistent, thereby highlighting the need for structured documentation templates and care pathways in the ED. The majority of patients presented within 24 hours of symptom onset, suggesting that dermatological issues are perceived by patients as urgent, even when not clinically severe. This further supports the need for efficient triage systems to avoid overburdening ED services with non-emergency cases while still capturing urgent conditions such as anaphylaxis, Stevens-Johnson Syndrome (SJS), and DRESS syndrome. However, the high proportion of patients presenting with non-emergent conditions underscores the potential for misallocation of ED resources and highlights the necessity for robust diagnostic protocols to differentiate urgent from non-urgent dermatological complaints [12]. This situation often leads to patients using emergency departments for issues that could be managed in primary care settings, potentially exacerbating ED overcrowding and resource strain [13,14].

Our study revealed that most clinical and demographic variables, including diagnosis, age group, gender, and symptom duration, did not show statistically significant associations with treatment or admission rates. This may reflect the heterogeneous nature of dermatological presentations in the ED, where clinical decision-making often relies on clinician gestalt and visible severity rather than structured criteria [15]. Our study revealed that most clinical and demographic variables, including diagnosis, age group, gender, and symptom duration, did not show statistically significant associations with treatment or admission rates. This may reflect the heterogeneous nature of dermatological presentations in the ED, where clinical decision-making often relies on clinician gestalt and visible severity rather than structured criteria [15]. This lack of clear correlation underscores the diagnostic challenges inherent in dermatology within an acute care setting, where many presentations are non-emergent and could potentially be managed in an outpatient capacity [1]. Conversely, the absence of an on-call dermatologist in many emergency departments could lead to suboptimal management of complex dermatological conditions, necessitating the development of clear referral pathways to specialised dermatology services [16].

Importantly, we observed a statistically significant association between the performance of investigations and the likelihood of documented follow-up (p < 0.001). This suggests that clinicians may be more likely to create follow-up plans for patients perceived as complex or uncertain, as indicated by ordering blood tests. This reinforces the value of protocols for structured assessment, as seen in prior audits [17].

This audit has notable strengths, including a clear methodology with defined inclusion and exclusion criteria, benchmarking against NICE and BAD guidance, and a comprehensive dataset spanning demographics, diagnoses, management, and outcomes. The findings are directly relevant to clinical practice, highlighting opportunities for protocol development, education, and improved follow-up.

Limitations include its single-centre and retrospective design, which restricts generalisability and makes the data dependent on documentation quality. The sample size (n = 80) limits statistical power, and the exclusion of urgent care referrals may underestimate the true burden of dermatology presentations. Qualitative insights into clinician decision-making and barriers to referral were also not captured. These factors mean the findings should be viewed as baseline data to inform local quality improvement and re-audit rather than definitive conclusions.

## Conclusions

This audit demonstrates that dermatological presentations to the adult emergency department are both frequent and clinically diverse, with allergic reactions and skin infections being the most common. Despite this, there is significant variability in the investigation, treatment, and documentation of such cases. A substantial proportion of patients received no follow-up or specialist referral, and adherence to national guidelines, particularly regarding timely antibiotic administration and comprehensive history-taking, was inconsistent.

These findings underscore the need for a more structured approach to managing dermatology-related complaints in emergency settings. The development of a local dermatology protocol, implementation of standardised documentation tools, and targeted education for ED staff are essential next steps. Such interventions may enhance clinical efficiency, reduce inappropriate admissions, and improve patient outcomes. Re-auditing following the implementation of these changes will be important to assess the impact and ensure continuous improvement in dermatological care in the emergency department.
